# Prognostic model on pregnancy outcomes for women with recurrent spontaneous abortions treated with cyclosporin A: A single-institution experience

**DOI:** 10.1016/j.clinsp.2024.100349

**Published:** 2024-04-12

**Authors:** Ze-Ming Zhang, Na Zhang, Xiao-Fei Wang

**Affiliations:** Department of Rheumatology, Shengjing Hospital of China Medical University, Shenyang, China

**Keywords:** Recurrent spontaneous abortion, Cyclosporin A, Pregnancy outcome, Prognostic factor, Retrospective

## Abstract

**Background:**

This study aimed to identify prognostic factors for pregnancy outcomes and construct a prognostic model for pregnancy outcomes in women with Recurrent Spontaneous Abortions (RSA) treated with cyclosporin A.

**Methods:**

A total of 154 RSA patients treated with cyclosporin A between October 2016 and October 2018 were retrospectively recruited. Multivariate logistic regression was applied to identify the prognostic factors for pregnancy success in RSA women treated with cyclosporin A. The Receiver Operating Characteristic (ROC) curve was applied to construct prognostic value, and the prognostic performance was assessed using area under the ROC.

**Results:**

After adjusting potential confounding factors, the authors noted increased age (OR = 0.771; 95 % CI 0.693‒0.858; *p* < 0.001) and positive antinuclear antibodies (OR = 0.204; 95 % CI 0.079‒0.526; *p* = 0.001) were associated with a reduced incidence of pregnancy success, while positive anti-β2 glycoprotein-I-antibody (OR = 21.941; 95 % CI 1.176‒409.281; *p =* 0.039) was associated with an increased incidence of pregnancy success after treated with cyclosporin A. The AUC of combining these variables for predicting pregnancy failure was 0.809 (95 % CI 0.735‒0.880).

**Conclusions:**

This study systematically identified the prognostic factors for pregnancy success in women treated with cyclosporin A, and the constructed prognostic model based on these factors with relatively higher prognostic value. Further large-scale prospective studies should be performed to validate the prognostic value of the constructed model.

## Highlights

• The prognostic model for pregnancy outcomes was constructed.
• The prognostic factors included age, ANA, and anti-β2 glycoprotein I antibody levels.
• The prognostic models showed higher value for predicting pregnancy success.

## Introduction

Recurrent Spontaneous Abortion (RSA) is defined as two or more spontaneous pregnancy losses before 20 weeks of gestation with the same sexual partner and is regarded as a common obstetric complication in the early pregnancy stage. ^1^ The morbidity of RSA ranges from 2–4 %, with 80 % of cases taking place in the first trimester.^2^ The pathogenesis of RSA occurrence in the first trimester is more complicated than that in the second trimester, and the etiologic factors remain unidentified in nearly 50 % of RSA cases occurring in the first trimester.^3^ Immune-related RSA can be divided into alloimmune and autoimmune types based on maternal immunological factors.^4^ The alloimmune type is defined as Unexplained RSA (URSA) and is related to an imbalance of maternal-fetal immune tolerance, which accounts for a nearly 50 % incidence rate in RSA.^5^ In contrast, autoimmune RSA is caused by autoimmune diseases and is significantly related to autoimmune factors.^6^

The balance between immune tolerance and effector immunity is significantly related to successful pregnancy, and an imbalance in the regulatory system can cause adverse reproductive outcomes. Cyclosporine A (CsA), isolated from metabolites of Trichoderma polysporum and Cladospora, can inhibit autoimmune responses.^7^ The use of CsA can regulate maternal-fetal immunity by inhibiting maternal immune rejection of embryonic antigens and promoting the growth, movement, and invasion of trophoblast cells, and thus it is considered an effective drug for the treatment of RSA.^8^ The use of conventional doses of prednisone and CsA in pregnant patients following organ transplantation has been established as safe for fetuses and newborns.^7^

Several studies have addressed the effects of CsA on pregnancy outcomes.^9^, ^10^, ^11^ A study by Li et al. found that low-dose CsA promotes the proliferation, invasion, and migration of villous trophoblast cells, allowing successful implantation to be achieved.^9^ Further, Azizi et al. found that the use of CsA in women with RSA with an elevated TH1/TH2 ratio was significantly associated with an elevated live birth rate and improved TH1/TH2 ratio.^10^ Moreover, the use of CsA has been shown to improve embryo implantation and pregnancy rates in patients undergoing in vitro fertilization and embryo transfer.^11^ However, the rate of failure in pregnancy outcomes for RSA women treated with CsA remains high, and the prognostic factors require identification for further management and improvement of pregnancy outcomes. Therefore, the current retrospective study was performed to identify the prognostic factors and construct prognostic model for pregnancy outcomes in RSA women treated with CsA.

## Materials and methods

### *Study design and patients*

This retrospective cohort study recruited 209 patients with RSA who were admitted to the present study's hospital between October 2016 and October 2018. This study was approved by the Ethics Committee of Shengjing Hospital of China Medical University (nº 2022PS014K), and informed consent was waived owing to the retrospective design of the current study. All patients were treated with CsA, with 25 mg bid per day from the preparation for pregnancy until 12 weeks of gestation or abortion. The preparation of pregnancy will be followed by a one-year pre-pregnancy phase, and if participants do not become pregnant within that year, their treatment and participation will be terminated. The additional inclusion criteria are as follows: 1) Continued spontaneous abortion or unexplained fetal arrest and death occurred in utero more than two times; and 2) Neither partner was consanguineous and had normal karyotypes. After removing patients with incomplete clinical data, 154 were ultimately selected for analysis.

### *Data collection*

The characteristics of the recruited patients were collected using a predefined questionnaire and medical records. Collected variables included age, disease status (Recurrent Spontaneous Abortion complicated with Rheumatism [RRSA] and URSA), Antinuclear Antibodies (ANA), Anti-Cardiolipin Antibodies (ACA), β2-glycoprotein, Lupus Anticoagulant (LA), anti-α-lined protein antibody, anti-Sjogren Syndrome A antibody (anti-SSA antibody), anti-Sjogren Syndrome B antibody (anti-SSB antibody), complement, Immunoglobulin (Ig), platelet aggregation rate, thrombelastogram, Antithrombin III activity (AT-IIIA), IL-2, IL-4, IL-6, IL-10, IL-17, IFN-γ, TNF-α, Th/Ts, Natural Killer Cell (NK), and B cell percentages. The RRSA was defined as RSA complicated with autoimmune diseases, including positive antinuclear antibodies, anti-thyroid antibodies, or antiphospholipid antibodies. The URSA was defined after removing other causes of RSA, including abnormalities of the uterus or cervix, chromosomal abnormalities, infections, endocrine and metabolic diseases, congenital thrombophilia, autoimmune diseases, and others.

### *Outcome definition*

Pregnancy success was defined as the occurrence of uneventful pregnancies that were spontaneously conceived, resulting in the delivery of a live baby following treatment. Pregnancy failure was defined as the termination of pregnancy and failure to deliver a live baby due to abortion, fetal arrest, fetal death, or other reasons following treatment.

### *Statistical analysis*

The characteristics of patients according to pregnancy outcome are shown as means (standard deviation) or medians (quartile) for continuous variables according to data distribution, while categorical variables are shown as events (frequency). The differences between groups were assessed using a *t*-test and the Kruskal-Wallis test for continuous variables, whereas the Chi-Square test was applied to assess the differences in categorical variables. Multivariate logistic regression was applied to identify the prognostic factors of pregnancy success in women treated with CsA. The area under the receiver operating characteristic curve (AUC) and 95 % Confidence Interval (95 % CI) were calculated to assess the prognostic values of the constructed model. Exploratory analyses were performed according to disease status. All reported p-values were 2-sided, and the inspection level was set at 0.05. Statistical analysis in this study was performed using SPSS version 19.0 (SPSS 19.0, IBM Corp., Armonk, NY, USA).

## Results

### *General characteristics*

A total of 154 patients were analyzed, with a mean age of 33.24 years. The characteristics of patients according to disease status are shown in Table 1. There were significant differences between URSA and RRSA for ANA (*p <* 0.001), anti-β2 glycoprotein-I-antibodies (*p =* 0.002), anti-SSA antibodies (*p <* 0.001), and anti-SSB antibody (*p =* 0.043). However, there were no significant differences between URSA and RRSA for ACA (*p =* 0.108), LA (*p =* 0.136), anti-α-lined protein antibody (*p =* 0.267), lower complement (*p =* 0.803), elevated Ig (*p =* 0.869), age (*p =* 0.466), platelet aggregation rate (*p =* 0.228), thrombelastogram (*p =* 0.315), AT-IIIA (*p =* 0.244), IL-2 (*p =* 0.144), IL-4 (*p =* 0.269), IL-6 (*p =* 0.504), IL-10 (*p =* 0.615), IL-17 (*p =* 0.299), INF-γ (*p =* 0.857), TNF-α (*p =* 0.758), Th/Ts (*p =* 0.104), NK (*p =* 0.961), B-cell percentage (*p =* 0.308) and pregnancy outcome (*p =* 0.302).

**Table 1:** The baseline characteristics of included patients according to disease status.

Variable	URSA (*n* = 117)	RRSA (*n =* 37)	*p*-value
ANA			< 0.001
Negative	96 (82.05)	18 (48.65)	
Positive	21 (17.95)	19 (51.35)	
ACA			0.108
Negative	112 (95.73)	32 (86.49)	
Positive	5 (4.27)	5 (13.51)	
Anti-β2 glycoprotein-I-antibodies			0.002
Negative	114 (97.44)	30 (81.08)	
Positive	3 (2.56)	7 (18.92)	
LA			0.136
Negative	79 (67.52)	20 (54.05)	
Positive	38 (32.48)	17 (45.95)	
Anti-α-lined protein antibody			0.267
Negative	84 (71.79)	23 (62.16)	
Positive	33 (28.21)	14 (37.84)	
Anti-SSA antibody			< 0.001
Negative	115 (98.29)	30 (81.08)	
Positive	2 (1.71)	7 (18.92)	
Anti-SSB antibody			0.043
Negative	116 (99.15)	34 (91.89)	
Positive	1 (0.85)	3 (8.11)	
Lower complement			0.803
No	105 (89.74)	32 (86.49)	
Yes	12 (10.26)	5 (13.51)	
Elevated Ig			0.869
No	111 (94.87)	36 (97.30)	
Yes	6 (5.13)	1 (2.70)	
Age (years)	33.00 (30.00, 36.00)	33.00 (30.00, 35.00)	0.466
Platelet aggregation rate	85.40 (76.70, 89.20)	84.70 (13.00, 88.00)	0.228
Thrombelastogram	5.00 (4.60, 5.60)	5.20 (4.60, 5.70)	0.315
AT_IIIA (%)	103.22 (10.62)	105.59 (11.17)	0.244
IL_2 (pg/mL)	1.41 (1.09, 1.74)	1.54 (1.36, 1.84)	0.144
IL_4 (pg/mL)	1.68 (1.35, 2.15)	1.93 (1.48, 2.37)	0.269
IL_6 (pg/mL)	2.14 (1.62, 2.94)	2.18 (1.83, 2.91)	0.504
IL_10 (pg/mL)	1.79 (1.53,2.11)	1.87 (1.56,2.13)	0.615
IL_17 (pg/mL)	7.62 (6.17, 9.71)	7.39 (5.46, 9.14)	0.299
INF-γ (pg/mL)	1.89 (1.53, 2.40)	1.96 (1.62, 2.34)	0.857
TNF-α (pg/mL)	2.31 (1.70,3.18)	2.38 (1.73,3.79)	0.758
Th/Ts	1.52 (1.19,2.10)	1.35 (1.01,1.87)	0.104
NK (%)	14.70 (11.50, 20.70)	16.20 (9.30, 21.80)	0.961
B cell percent (%)	11.70 (9.80, 14.90)	13.50 (10.00, 15.80)	0.308
Pregnancy outcome			0.302
Failure	39 (33.33)	9 (24.32)	
Success	78 (66.67)	28 (75.68)	

### *Prognostic factors for pregnancy success after CsA treatment*

**Fig. 1:**
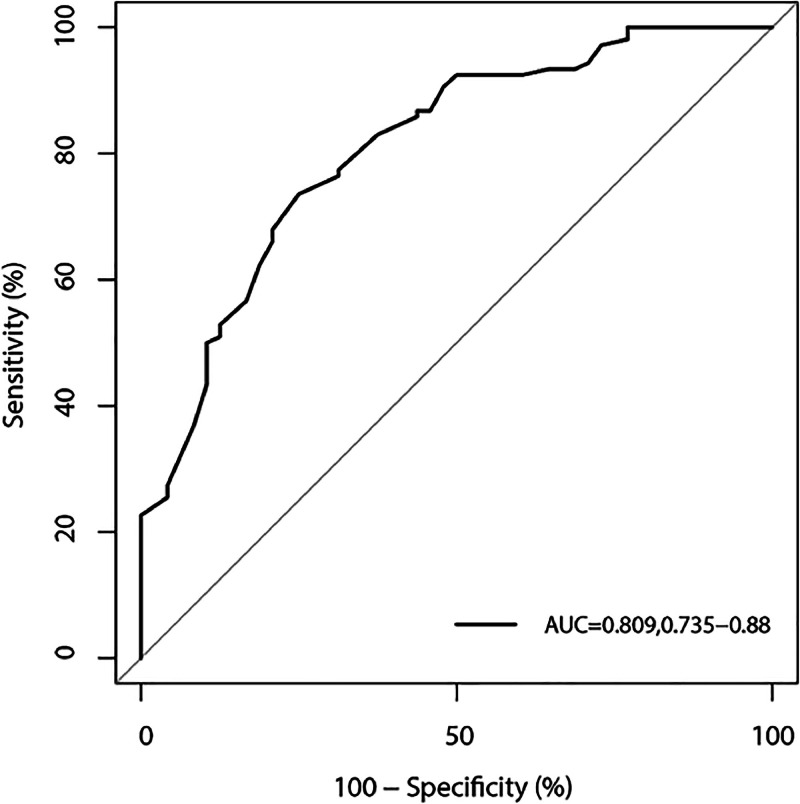
The area under the receiver operating characteristic curve for predicting pregnancy success.

The prognostic factors identified for pregnancy success after CsA treatment using multivariate logistic regression are shown in Table 2. When the full model logistic regression was performed, the authors noted the pregnancy success could affected by increased age (OR = 0.728; 95 % CI 0.636‒0.833; *p <* 0.001), positive ANA (OR = 0.074; 95 % CI 0.019‒0.287; *p <* 0.001), positive ACA (OR = 0.131; 95 % CI 0.020‒0.845; *p =* 0.033), and anti-β2 glycoprotein-I-antibody (OR = 28.308; 95 % CI 2.023‒396.114; *p =* 0.013). When applied step-wise logistic regression, the authors noted that increased age (OR = 0.771; 95 % CI 0.693‒0.858; *p <* 0.001) and positive ANA (OR = 0.204; 95 % CI 0.079‒0.526; *p =* 0.001) were associated with a reduced incidence of pregnancy success, whereas positive anti-β2 glycoprotein-I-antibody was associated with an increased incidence of pregnancy success (OR = 21.941; 95 % CI 1.176‒409.281; *p =* 0.039). The AUC of combining these variables in stepwise logistic regression for predicting pregnancy success was 0.809 (95 % CI 0.735‒0.880); (Fig. 1).

**Table 2:** Logistic regression for the prognostic factors for pregnancy success.

Variable	Full mode		Step wise	
	OR (95 % CI)	*p*‒value	OR (95 % CI)	*p*-value
Age (years)	0.728 (0.636‒0.833)	<0.001	0.771 (0.693‒0.858)	<0.001
ANA	0.074 (0.019‒0.287)	<0.001	0.204 (0.079‒0.526)	0.001
ACA	0.131 (0.020‒0.845)	0.033	0.188 (0.034‒1.049)	0.057
Anti-β2 glycoprotein-I-antibodies	28.308 (2.023‒396.114)	0.013	21.941 (1.176‒409.281)	0.039
LA	1.275 (0.448‒3.624)	0.649		
Anti-α-lined protein antibody	1.293 (0.456‒3.669)	0.629		
Anti-SSA antibody	3.228 (0.347‒29.992)	0.303		
Anti-SSB antibody	12.173 (0.188‒789.978)	0.240		
Lower complement	1.930 (0.362‒10.294)	0.441		
Elevated Ig	0.129 (0.014‒1.179)	0.070	0.202 (0.032‒1.292)	0.091
Platelet aggregation rate	0.990 (0.976‒1.005)	0.192		
Thrombelastogram	1.451 (0.815‒2.583)	0.206		
AT_IIIA (%)	0.999(0.954‒1.045)	0.957		
IL_2 (pg/mL)	0.639 (0.369‒1.105)	0.109		
IL_4 (pg/mL)	1.166 (0.571‒2.383)	0.674		
IL_6 (pg/mL)	1.066 (0.723‒1.570)	0.748		
IL_10 (pg/mL)	1.140 (0.412‒3.159)	0.801		
IL_17 (pg/mL)	0.981 (0.817‒1.179)	0.842		
INF-γ (pg/mL)	1.630 (0.946‒2.807)	0.078		
TNF-α (pg/mL)	1.134 (0.873‒1.474)	0.345		
Th/Ts	1.605 (0.742‒3.469)	0.229		
NK (%)	0.948 (0.882‒1.019)	0.149		
B cell percent (%)	0.948 (0.852‒1.055)	0.325

### *The prognostic factors for pregnancy success in URSA and RRSA*

The pregnancy success rate following treatment with CsA in RRSA patients was higher than that in URSA patients (75.68 % vs. 66.67 %), while the difference in this value between patients with RRSA and URSA was not statistically significant.

The characteristics of the included URSA patients according to pregnancy outcomes are shown in Table S1. There were significant differences between pregnancy failure and pregnancy success in URSA patients with respect to age (*p <* 0.001) and ANA (*p =* 0.002), while no other significant differences between groups were observed. Moreover, multivariate logistic regression analysis suggested that increased age (OR = 0.809; 95 % CI 0.725‒0.902; *p <* 0.001), positive ANA (OR = 0.158; 95 % CI 0.051‒0.487; *p =* 0.001), and positive ACA (OR = 0.089; 95 % CI 0.010‒0.797; *p =* 0.001) were associated with a reduced incidence of pregnancy success (Table S2). The AUC of combining these variables for predicting pregnancy success in patients with URSA was 0.779 (95 % CI 0.689‒0.870); (Fig. S1).

The characteristics of the included RRSA patients according to pregnancy outcomes are shown in Table S3. The pregnancy failure group was significantly older than the pregnancy success group (*p =* 0.004), while no other significant difference was observed. Moreover, multivariate logistic regression analysis indicated that older age was associated with a lower incidence of pregnancy success (OR = 0.683; 95 % CI 0.512‒0.910; *p =* 0.009; Table S4). The AUC of combining these variables for predicting pregnancy success in RRSA patients was 0.823 (95 % CI 0.655‒0.990); (Fig. S2).

In the planning stage, changes in NK should be analyzed for patients before and after treatment with CsA. However, only 49 patients reported NK before and after CsA treatment. The percentage of NK cells before and after treatment with CsA was 20.31 % and 15.38 %, respectively, and the difference was statistically significant (−4.93 %; *p <* 0.05).

## Discussion

CsA is widely used as an immunosuppressant for organ transplantation and autoimmune diseases. Animal studies have shown that low doses can induce maternal-fetal immune tolerance and enhance trophoblast invasion. CsA is used to prevent and resist graft-vs.-host reactions and no adverse reactions to fetuses have been found in pregnant patients who take conventional doses of CsA for an extended time following organ transplantation.^12^ Previous studies have addressed the treatment effects of CsA in women with RSA.^11^^,^^13^ For example, Fu et al. recruited 168 refractory immune RSA patients treated with CsA (2‒4 mg/kg), and maintenance of 80‒150 mg/L CsA was associated with an elevated live birth rate.^13^ Ling et al. identified 86 URSA patients treated with 100 mg/day for 12 weeks, which increased the live birth rate, and no adverse events were detected.^11^ These results suggest that CsA should be considered an effective treatment for URSA. However, the prognostic factors for pregnancy success in women taking CsA remain controversial. The current retrospective study recruited 154 women treated with CsA, and the characteristics of the included patients varied widely. The pregnancy success rate in RSA patients treated with CsA was 68.83 %, and the incidence of pregnancy success can be affected by age, ANA, and anti-β2 glycoprotein I antibody, and the prognostic performance of the constructed model based on these factors was relatively higher. Subgroup analyses found the prognostic factors for pregnancy success in URSA women included age, positive ANA, and positive ACA, and the prognostic value of the prognostic model based on these factors was moderate. Moreover, the prognostic factors for pregnancy success in RRSA women included increased age, and the prognostic value was higher for predicting further pregnancy success.

Several studies have previously addressed the prognostic factors of pregnancy outcomes in patients with RSA.^14^^,^^15^ One retrospective study by Yang et al. recruited 492 singleton pregnant women and found that a history of first-trimester RSA was an independent risk factor for cesarean section and pregnancy complications.^14^ Additionally, Liu et al. conducted a retrospective study of 1240 pregnant women with a history of RSA and found that a cutoff value of serum β-subunit of human chorionic gonadotropin of 88,000 IU/L could predict early pregnancy outcomes.^15^ Maesawa et al. recruited 175 women with RSA and found that two or more biochemical pregnancies were associated with an increased risk of reproductive failure and spontaneous abortion with normal chromosomes, together with a lower chance of live birth.^16^ In another study, Caetano et al. performed a case-control study and found that age > 40-years, immunological factors, and two or more concomitant factors were significantly related to poor gestational outcomes in women.^17^ Kruse et al. recruited 217 women with URSA and found that low maternal serum mannan-binding lectin levels could affect pregnancy outcomes for women with URSA.^18^ However, the prognostic factors for pregnancy success in women with RSA treated with CsA have not yet been identified. Therefore, the authors performed this retrospective study to identify prognostic factors for pregnancy outcomes for RSA women treated with CsA.

Increased age and ANA positivity were associated with a reduced incidence of pregnancy success in this study, which aligns with the existing evidence that maternal age is an independent predictor of poor maternal and fetal outcomes during pregnancy.^19^ The prevalence of ANA has previously been demonstrated to be higher in recurrent pregnancy loss, and positive ANA results are associated with an increased risk of miscarriage.^20^ Moreover, the disappearance of ANA in early pregnancy is associated with ongoing pregnancy, which indicates an improvement in systemic autoimmunity.^21^ The pregnancy success group presented with higher positive anti-β2 glycoprotein-I-antibody rates in this study. As β2-glycoprotein functions as a physiologic anticoagulant by inhibiting the key procoagulant activities of thrombin,^22^ pregnancy outcomes could be affected. The results of the present study found that positive anti-β2 glycoprotein-I-antibody patients treated with CsA were associated with an increased incidence of pregnancy success, which suggests the use of CsA could offer more benefit for patients who presented positive anti-β2 glycoprotein-I-antibody. The result might not have been stable owing to the marginal 95 % CI, and further prospective studies are needed to verify this finding.

Another study previously found that an imbalance of Th/Ts plays an important role in the progression of RSA.^23^ Villous trophoblast cells at the maternal/fetal interface initiate and complete uterine placental circulation, secreting a variety of cytokines to maintain the Th1 /Th2 cell balance. Once this balance is disrupted, the fetus is attacked and miscarried.^24^ IFN-γ, IL4, IL13 and other cytokines secreted by Th1 and Th2 cells interact with each other to maintain maternal immune tolerance to the fetus and ensure fetal growth and development during pregnancy, while Th1 cells oversecrete IFN-γ, which can damage trophoblast cells by inducing activated NK cells, hindering embryo implantation and placental growth and development, and thus lead to abortion.^25^ The use of CsA may restore the balance of Th/Ts, which could explain why Th/Ts was not associated with the incidence of pregnancy success in the present study.^26^

Several limitations of this study should be acknowledged. First, it was designed as a retrospective cohort, and the results could, therefore, have been affected by selection and recall biases. Second, the background treatment strategies for RSA patients differ, which could affect the pregnancy outcomes. Third, the severity of RSA could affect pregnancy outcomes, which were not addressed in this study. Fourth, it remains unclear whether the RSA is caused by alloimmunological factors, which should be further explored. Finally, stratified analyses were only performed based on disease status, whereas based on several other important factors were not performed.

## Conclusions

The findings of this study showed that age, ANA, and anti-β2 glycoprotein I antibody levels were significantly associated with the incidence of pregnancy success, and the prognostic models for these variables showed higher value for predicting pregnancy success in women treated with CsA. Further large-scale prospective studies should be performed to verify the results of this study and validate the constructed prognostic model.

## Funding

This study was not funded by any organization.

## Data Sharing

The datasets used and/or analyzed during the current study are available from the corresponding author upon reasonable request.

## CRediT authorship contribution statement

**Ze-Ming Zhang:** Data curation, Formal analysis, Investigation, Methodology, Validation, Writing – original draft. **Na Zhang:** Investigation, Methodology, Writing – review & editing. **Xiao-Fei Wang:** Conceptualization, Methodology, Project administration, Resources, Supervision, Validation, Writing – review & editing.

## Declaration of competing interest

The authors declare no conflicts of interest.

## Appendix Supplementary materials

Image, application 1
