# Supplementary material for: Prognostic model on pregnancy outcomes for women with recurrent spontaneous abortions treated with cyclosporin A: A single-institution experience
Source: Clinics (Sao Paulo). 2024 Apr 12;79:100349. doi: 10.1016/j.clinsp.2024.100349 (PMC11033085; doi:10.1016/j.clinsp.2024.100349)

**CLINICS-D-23-00463_Supplementary Material**

**Table S1** The baseline characteristics of included URSA patients according to pregnancy outcome.

| **Variable** | **Pregnancy failure**  **(n = 39)** | **Pregnancy success**  **(n = 78)** | **p-value** |
| --- | --- | --- | --- |
| Age (years) | 35.64 (4.65) | 32.15 (4.38) | <0.001 |
| ANA |  |  | 0.002 |
| Negative | 26 (66.67) | 70 (89.74) |  |
| Positive | 13 (33.33) | 8 (10.26) |  |
| ACA |  |  | 0.419 |
| Negative | 36 (92.31) | 76 (97.44) |  |
| Positive | 3 (7.69) | 2 (2.56) |  |
| anti-β2 glycoprotein-I-antibodies |  |  | 0.535 |
| Negative | 39 (100.00) | 75 (96.15) |  |
| Positive | 0 (0.00) | 3 (3.85) |  |
| LA |  |  | 0.264 |
| Negative | 29 (74.36) | 50 (64.10) |  |
| Positive | 10 (25.64) | 28 (35.90) |  |
| Anti-α-lined protein antibody |  |  | 0.663 |
| Negative | 29 (74.36) | 55 (70.51) |  |
| Positive | 10 (25.64) | 23 (29.49) |  |
| Anti-SSA antibody |  |  | 1.000 |
| Negative | 38 (97.44) | 77 (98.72) |  |
| Positive | 1 (2.56) | 1 (1.28) |  |
| Anti-SSB antibody |  |  | 1.000 |
| Negative | 39 (100.00) | 77 (98.72) |  |
| Positive | 0 (0.00) | 1 (1.28) |  |
| Lower complement |  |  | 0.747 |
| No | 36 (92.31) | 69 (88.46) |  |
| Yes | 3 (7.69) | 9 (11.54) |  |
| Elevated Ig |  |  | 0.657 |
| No | 36 (92.31) | 75 (96.15) |  |
| Yes | 3 (7.69) | 3 (3.85) |  |
| Platelet aggregation rate | 86.90 (80.40, 89.50) | 84.70 (12.80, 88.40) | 0.203 |
| [Thrombelastogram](javascript:;) | 4.90 (4.40, 5.30) | 5.00 (4.70, 5.80) | 0.261 |
| AT_IIIA (%) | 104.08 (10.65) | 102.79 (10.65) | 0.541 |
| IL_2 (pg/mL) | 1.42 (1.04, 1.77) | 1.40 (1.18, 1.74) | 0.397 |
| IL_4 (pg/mL) | 1.60 (1.20, 2.29) | 1.69(1.38, 2.15) | 0.744 |
| IL_6 (pg/mL) | 2.20 (1.69, 2.82) | 2.06 (1.62, 3.10) | 0.936 |
| IL_10 (pg/mL) | 1.71 (1.47, 2.06) | 1.83 (1.54, 2.13) | 0.268 |
| IL_17 (pg/mL) | 7.80 (5.76, 9.37) | 7.54 (6.22, 9.83) | 0.716 |
| INF-γ (pg/mL) | 1.86 (1.63, 2.40) | 1.90 (1.51, 2.40) | 0.952 |
| TNF-α (pg/mL) | 2.32 (1.54, 3.18) | 2.28 (1.79, 3.24) | 0.751 |
| Th/Ts | 1.65 (1.30, 2.10) | 1.48 (1.16, 2.10) | 0.408 |
| NK (%) | 17.50 (10.80, 21.40) | 14.20 (11.60, 20.70) | 0.532 |
| B cell percent (%) | 12.00 (9.30, 14.90) | 11.60 (10.00, 15.00) | 0.910 |

**Table S2** Logistic regression for the prognostic factors for pregnancy success in patients with URSA.

| **Variable** | **Full mode** | | | **Step wise** | | |
| --- | --- | --- | --- | --- | --- | --- |
|  | **β** | **OR (95% CI)** | **p-value** | **β** | **OR (95% CI)** | **p-value** |
| Age (years) | -0.3151 | 0.730 (0.625‒0.852) | <0.001 | -0.2124 | 0.809 (0.725‒0.902) | <0.001 |
| ANA | -2.8513 | 0.058 (0.011‒0.302) | <0.001 | -1.8460 | 0.158 (0.051‒0.487) | 0.001 |
| ACA | -2.2693 | 0.103 (0.008‒1.419) | 0.089 | -2.4209 | 0.089 (0.010‒0.797) | 0.031 |
| anti-β2 glycoprotein-I-antibodies | 14.8059 | 2692400.60 (0.000‒I) | 0.970 |  |  |  |
| LA | -0.0778 | 0.925 (0.262‒3.263) | 0.904 |  |  |  |
| Anti-α-lined protein antibody | 0.1785 | 1.195 (0.351‒4.072) | 0.775 |  |  |  |
| Anti-SSA antibody | -0.3907 | 0.677 (0.016‒29.215) | 0.839 |  |  |  |
| Anti-SSB antibody | 9.9417 | 20778.792 (0.000‒I) | 0.990 |  |  |  |
| Lower complement | 0.7127 | 2.040 (0.239‒17.378) | 0.514 |  |  |  |
| Elevated Ig | -1.9088 | 0.148 (0.013‒1.710) | 0.126 |  |  |  |
| Platelet aggregation rate | -0.0121 | 0.988 (0.971‒1.005) | 0.171 |  |  |  |
| [Thrombelastogram](javascript:;) | 0.4925 | 1.636 (0.863‒3.104) | 0.132 |  |  |  |
| AT_IIIA (%) | -0.0088 | 0.991 (0.941‒1.044) | 0.742 |  |  |  |
| IL_2 (pg/mL) | -0.3636 | 0.695 (0.358‒1.350) | 0.283 |  |  |  |
| IL_4 (pg/mL) | -0.0684 | 0.934 (0.364‒2.399) | 0.887 |  |  |  |
| IL_6 (pg/mL) | 0.0657 | 1.068 (0.710‒1.607) | 0.752 |  |  |  |
| IL_10 (pg/mL) | 0.1609 | 1.175 (0.351‒3.926) | 0.794 |  |  |  |
| IL_17 (pg/mL) | 0.0496 | 1.051 (0.855‒1.291) | 0.637 |  |  |  |
| INF-γ (pg/mL) | 0.4943 | 1.639 (0.869‒3.094) | 0.127 |  |  |  |
| TNF-α (pg/mL) | 0.1324 | 1.142 (0.820‒1.589) | 0.433 |  |  |  |
| Th/Ts | 0.7107 | 2.035 (0.839‒4.940) | 0.116 |  |  |  |
| NK (%) | -0.0797 | 0.923 (0.847‒1.007) | 0.072 |  |  |  |
| B cell percent (%) | -0.0777 | 0.925 (0.821‒1.042) | 0.202 |  |  |  |

**Table S3** The baseline characteristics of included RRSA patients according to pregnancy outcome.

| **Variable** | **Pregnancy failure (n=9)** | **Pregnancy success (n=28)** | **p-value** |
| --- | --- | --- | --- |
| Age (years) | 38.00 (33.00, 40.00) | 31.00 (29.00, 34.50) | 0.004 |
| ANA |  |  | 0.447 |
| Negative | 3 (33.33) | 15 (53.57) |  |
| Positive | 6 (66.67) | 13 (46.43) |  |
| ACA |  |  | 1.000 |
| Negative | 8 (88.89) | 24 (85.71) |  |
| Positive | 1 (11.11) | 4 (14.29) |  |
| β2-[glycoprotein](javascript:;) |  |  | 0.656 |
| Negative | 8 (88.89) | 22 (78.57) |  |
| Positive | 1 (11.11) | 6 (21.43) |  |
| LA |  |  | 0.462 |
| Negative | 6 (66.67) | 14 (50.00) |  |
| Positive | 3 (33.33) | 14 (50.00) |  |
| Anti-α-lined protein antibody |  |  | 0.705 |
| Negative | 5 (55.56) | 18 (64.29) |  |
| Positive | 4 (44.44) | 10 (35.71) |  |
| Anti-SSA antibody |  |  | 0.656 |
| Negative | 8 (88.89) | 22(78.57) |  |
| Positive | 1 (11.11) | 6 (21.43) |  |
| Anti-SSB antibody |  |  | 1.000 |
| Negative | 8 (88.89) | 26 (92.86) |  |
| Positive | 1 (11.11) | 2 (7.14) |  |
| Lower complement |  |  | 1.000 |
| No | 8 (88.89) | 24 (85.71) |  |
| Yes | 1 (11.11) | 4 (14.29) |  |
| Elevated Ig |  |  | 0.243 |
| No | 8 (88.89) | 28 (100.00) |  |
| Yes | 1 (11.11) | 0 (0.00) |  |
| Platelet aggregation rate | 74.60 (11.80, 88.10) | 84.75 (40.10, 87.85) | 0.645 |
| [Thrombelastogram](javascript:;) | 5.26 (0.63) | 5.26 (0.88) | 0.978 |
| AT_IIIA (%) | 103.56 (7.83) | 106.25 (12.10) | 0.537 |
| IL_2 (pg/mL) | 1.82 (1.44, 2.13) | 1.52 (1.36, 1.74) | 0.235 |
| IL_4 (pg/mL) | 1.93 (1.56, 2.27) | 1.88 (1.35, 2.39) | 0.887 |
| IL_6 (pg/mL) | 2.18 (1.97, 2.37) | 2.27 (1.71, 3.20) | 0.915 |
| IL_10 (pg/mL) | 1.84 (1.35, 1.91) | 1.94 (1.57, 2.20) | 0.202 |
| IL_17 (pg/mL) | 8.23 (5.98, 8.94) | 7.16 (5.31, 9.28) | 0.348 |
| INF-γ (pg/mL) | 2.09 (0.70) | 1.99(0.63) | 0.676 |
| TNF-α (pg/mL) | 1.94 (1.57, 2.59) | 2.45 (1.79, 4.14) | 0.173 |
| Th/Ts | 1.57 (0.59) | 1.43 (0.48) | 0.494 |
| NK (%) | 19.41 (10.33) | 15.82 (7.72) | 0.271 |
| B cell percent (%) | 11.77 (3.98) | 13.68 (4.03) | 0.224 |

**Table S4** Logistic regression for the prognostic factors for pregnancy success in patients with RRSA.

| **Variable** | **Full mode** | | | **Step wise** | | |
| --- | --- | --- | --- | --- | --- | --- |
|  | **β** | **OR (95% CI)** | **p-value** | **β** | **OR (95% CI)** | **p-value** |
| Age (years) | -2.4917 | 0.083 (0.000‒9.53485E22) | 0.930 | -0.3818 | 0.683(0.512‒0.910) | 0.009 |
| ANA | -12.2174 | 0.000 (0.000‒I) | 0.988 |  |  |  |
| ACA | 4.9443 | 140.368 (0.000‒2.3823E216) | 0.984 |  |  |  |
| anti-β2 glycoprotein-I-antibodies | 3.2972 | 27.037 (0.000‒2.4276E238) | 0.991 |  |  |  |
| LA | 0.0271 | 1.027 (0.000‒8.4209E163) | 1.000 |  |  |  |
| Anti-α-lined protein antibody | -14.0571 | 0.000 (0.000‒I) | 0.978 |  |  |  |
| Anti-SSA antibody | 9.5954 | 14696.402 (0.000‒I) | 0.984 |  |  |  |
| Anti-SSB antibody | -69.7694 | 0.000 (0.000‒I) | 0.931 |  |  |  |
| Lower complement | 16.6585 | 17166429.3 (0.000‒I) | 0.978 |  |  |  |
| Elevated Ig | 73.5585 | 8.83201E31 (0.000‒I) | 0.982 |  |  |  |
| Platelet aggregation rate | 0.1384 | 1.148 (0.008‒159.780) | 0.956 |  |  |  |
| [Thrombelastogram](javascript:;) | -4.0542 | 0.017 (0.000‒2.3573E138) | 0.980 |  |  |  |
| AT_IIIA (%) | 0.6633 | 1.941 (0.000‒9.12577E10) | 0.958 |  |  |  |
| IL_2 (pg/mL) | -6.4931 | 0.002 (0.000‒I) | 0.989 |  |  |  |
| IL_4 (pg/mL) | 10.3936 | 32649.355 (0.000‒1.0384E137) | 0.947 |  |  |  |
| IL_6 (pg/mL) | 5.9254 | 374.445 (0.000‒8.9771E240) | 0.983 |  |  |  |
| IL_10 (pg/mL) | 7.2395 | 1393.447 (0.000‒I) | 0.985 |  |  |  |
| IL_17 (pg/mL) | 0.0915 | 1.096 (0.000‒5.76511E48) | 0.999 |  |  |  |
| INF-γ (pg/mL) | -17.1511 | 0.000 (0.000‒2.1538E101) | 0.893 |  |  |  |
| TNF-α (pg/mL) | 0.9272 | 2.527 (0.000‒1.29614E19) | 0.966 |  |  |  |
| Th/Ts | 3.3309 | 27.964 (0.000‒7.8872E192) | 0.988 |  |  |  |
| NK (%) | 0.2821 | 1.326 (0.000‒1.47088E16) | 0.988 |  |  |  |
| B cell percent (%) | 0.6372 | 1.891 (0.000‒1.53877E51) | 0.992 |  |  |  |

**Figure S1** The AUC of combined these variables for predicting pregnancy success in patients with URSA.


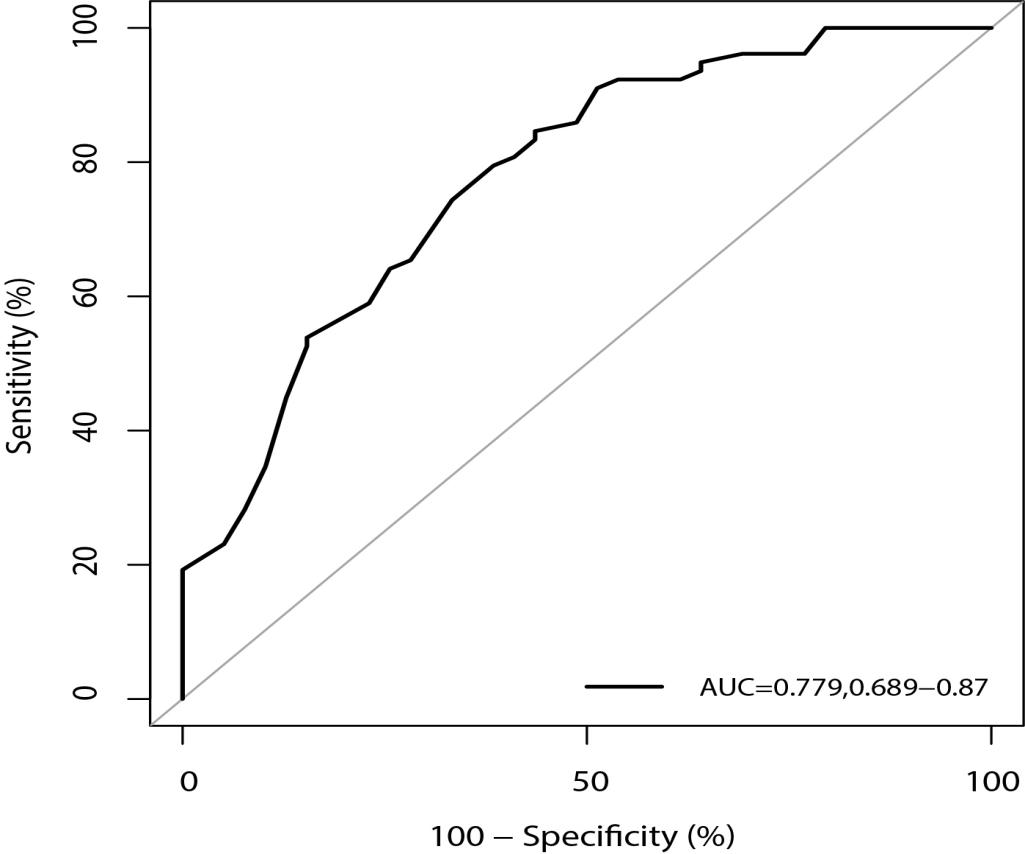


**Figure S2** The AUC of combined these variables for predicting pregnancy success in patients with RRSA


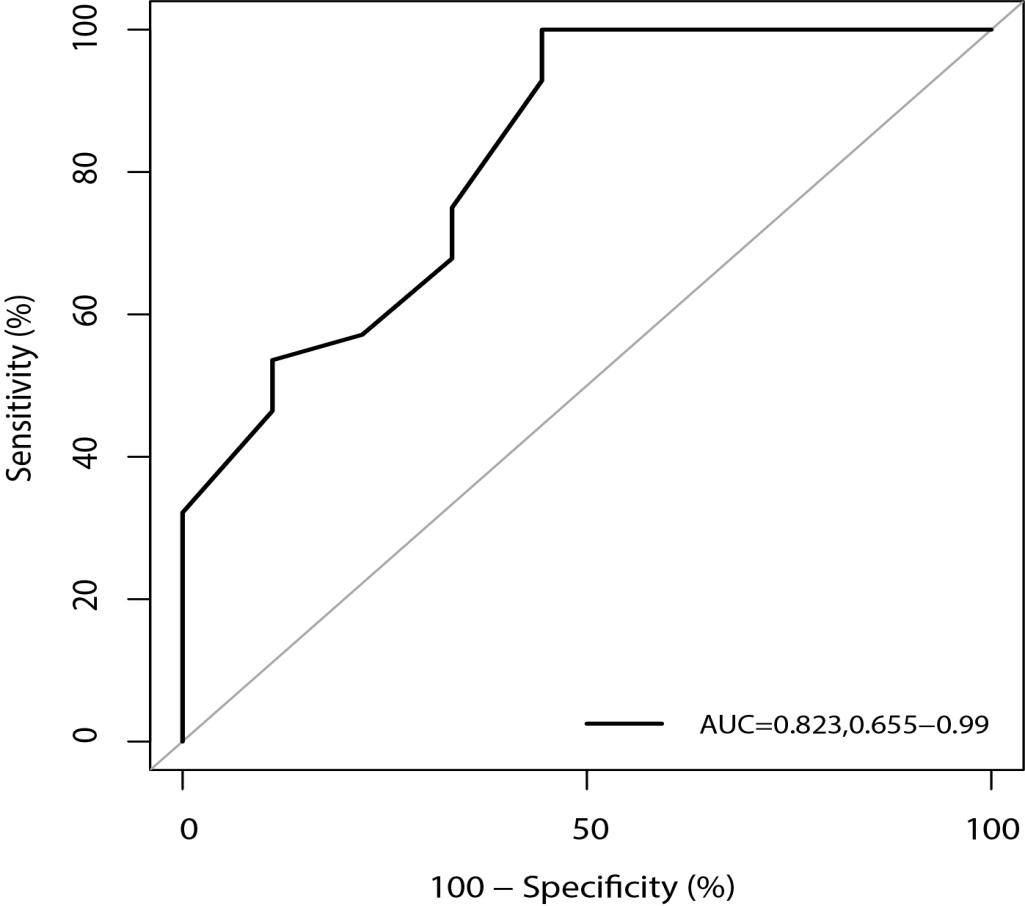

Supplement: Supplementary file 1 [file mmc1.docx]
